# The roles of long non-coding RNAs in Alzheimer's disease diagnosis, treatment, and their involvement in Alzheimer's disease immune responses

**DOI:** 10.1016/j.ncrna.2024.03.008

**Published:** 2024-03-16

**Authors:** Xiaoben Wu, Pengcheng Xia, Lei Yang, Chao Lu, Zhiming Lu

**Affiliations:** a Department of Clinical Laboratory, Shandong Provincial Hospital Affiliated to Shandong First Medical University, Jinan, China; b Department of Medical Engineering, Shandong Provincial Hospital Affiliated to Shandong First Medical University, Jinan, China

**Keywords:** Alzheimer's disease, Long non-coding RNA, Diagnosis, Treatment, Progression

## Abstract

Alzheimer's disease (AD) is the most frequent type of dementia, presenting a substantial danger to the health and well-being of the aged population. It has arisen as a significant public health problem with considerable socioeconomic repercussions. Unfortunately, no effective treatments or diagnostic tools are available for Alzheimer's disease. Despite substantial studies on the pathophysiology of Alzheimer's, the molecular pathways underpinning its development remain poorly understood. Long non-coding RNAs (lncRNAs) vary in size from 200 nucleotides to over 100 kilobytes and have been found to play critical roles in various vital biological processes that play critical in developing Alzheimer's disease. This review intends to examine the functions of long non-coding RNAs in diagnosing and treating Alzheimer's disease and their participation in immunological responses associated with AD.

## 1 Background

Alzheimer's disease (AD), the most common cause of dementia, is a neurological disorder that worsens with time [1]. AD is characterized by memory loss and deficits in thinking, language, and problem-solving abilities. Alzheimer's disease affects around 6.5 million Americans aged 65 and above today. Without pharmaceutical advances to prevent, treat, or cure Alzheimer's disease, this amount might rise to 13.8 million by 2060 [2]. AD has been declared the sixth-leading cause of death in the United States in 2019 and the seventh-leading cause of death in 2020. In the United States, Alzheimer's disease is still the sixth leading cause of death among those aged 65 and over. Between 2000 and 2019, reported deaths from stroke, heart disease, and HIV decreased, whereas Alzheimer's mortality increased by more than 145% [3]. Men face a 19-29% lower likelihood of developing Alzheimer's disease compared to women [4]. In 2010, the countries with the highest incidence of individuals experiencing dementia, in descending order, were China, the USA, India, Japan, Germany, Russia, France, and Brazil, and the numbers surpassed one million for each of these countries [5]. Most long non-coding RNA (lncRNA) types are transcribed by RNA polymerase II (Pol II). Consequently, many possess 5′-end m^7^G caps and 3′-end poly(A) tails, suggesting that they undergo transcription and processing like messenger RNAs (mRNAs). LncRNAs are divided into various categories according to their characteristics [6]. According to where they originate in the genome, lncRNAs, for instance, may be divided into five categories: sense, antisense, bidirectional, intronic, and intergenic. They may be categorized into three groups according to their function: rRNA, tRNA, and cRNA. LncRNAs are classified as nuclear, cytoplasmic, or mitochondrial, depending on where they are located in the cell [7]. LncRNAs vary in size from 200 nucleotides to over 100 kilobytes and have been found to play critical roles in AD; for example, they have been found to regulate different gene expressions in AD and other diseases [8]. In Alzheimer's disease, there have been reports suggesting interaction between lncRNAs and histone modification, namely H3K4me3 and H3K27me3, and those implications are extended to be involved in the regulation of various genes [9]; in addition, these lncRNAs also work as epigenetic regulators by altering both histone methylation and DNA modification to control gene expression [10]. Furthermore, it has been found that lncRNAs may interact epigenetically with miRNAs to regulate mRNA stability, which is altered in AD [11]. On the other side, researchers have demonstrated that lncRNAs play a significant role in the development of AD by affecting both nuclear and cytoplasmic activities, and various dysregulated of lncRNAs have been associated with AD pathogenesis, with particular lncRNAs such as BACE1-AS, NEAT1, MALAT1, and SNHG1 [12]. These long non-coding RNAs are involved in various AD-related processes, including neurogenesis, synaptic dysfunction, amyloid beta buildup, neuroinflammation, insulin resistance, and glucose dysregulation [13]. Furthermore, lncRNAs regulate gene expression in the central nervous system, and dysregulation may lead to neuronal death in neurodegenerative disorders. Also, various studies on AD model mice revealed lncRNA-associated competing endogenous RNA networks, offering insights into putative processes underpinning AD pathogenesis [14].LncRNAs have also been shown to play a vital role in AD by functioning as miRNA sponges, and this function reduces the regulatory influence of microRNAs on messenger RNAs linked to AD [15,16]. The relationship mentioned above adds complexity to the miRNA-target network in AD, as discussed by Paraskevopoulou in 2016(15).In addition, experiments have shown that lncRNAs act as miRNA sponges and have a significant impact on AD(16), and studying endogenous miRNA sponges, especially lncRNAs, has offered vital insights into their potential and proven roles in AD [17,18]. Besides, in AD research, a significant aspect to consider is the possible impact of lncRNAs on nuclear splicing control, a crucial mechanism for creating proteome diversity and directing gene expression [19]; however, this has not been intensely exploited; thus, this aspect of lncRNA participation is of particular interest to researchers focused on gaining a thorough knowledge of AD.Furthermore, lncRNAs are gaining prominence as significant contributors within the complex molecular framework of Alzheimer's disease (AD) [20]. They are involved in several cellular mechanisms and are progressively acknowledged for their regulatory functions in the development and progression of AD(20). Multiple studies have been conducted that have discovered dysregulated expression patterns of particular lncRNAs in the brains of individuals with AD, suggesting their potential role in the course of the illness [21]. It is worth mentioning that some lncRNAs have been associated with significant elements of AD pathology, such as the accumulation of amyloid beta (Aβ), excessive phosphorylation of tau proteins, and neuroinflammation [22,23]. The regulatory impact of long non-coding RNAs (lncRNAs) on gene expression networks related to Alzheimer's disease (AD) is underscored by the dynamic interplay they have with epigenetic changes [24].Consequently, many lncRNAs have demonstrated promise as diagnostic or prognostic biomarkers for Alzheimer's disease (AD), in addition to their participation in pathogenic pathways [25]. The modified patterns of gene expression in peripheral blood or cerebral fluid have the potential to function as markers of illness development and contribute to the early identification of the condition. Furthermore, altering specific lncRNAs has excellent potential as a viable therapeutic intervention in Alzheimer's disease (AD) [26]. The possible modification of abnormal gene expression patterns identified in Alzheimer's disease by targeting these molecules presents innovative options for minimizing cognitive loss and resolving the molecular intricacies associated with the pathogenesis of this condition [27,28]. Similarly, new findings show that lncRNAs play a role in a wide range of neurological disorders, notably epilepsy, neurodegenerative illnesses such as Alzheimer's, and many other critical genetic disorders [29]. This review has the main target of reviewing the roles of long non-coding RNAs in Alzheimer's disease diagnosis, treatment and their involvement in Alzheimer's disease immune responses.

## 2 AD pathophysiology

Amyloid plaques, which form due to abnormal proteolytic processing of APP outside of cells, and NFT, which include as a result of hyperphosphorylated tau protein buildup within neurons, are pathological features of AD [30]. Amyloid plaques are mostly made up of amyloid (Aβ) peptides, particularly the (Aβ) 42 isoform, a very poisonous hydrophobic peptide [31]. The Aβ peptide's hydrophobic nature causes aggregation and the creation of the amyloid plaque core, which adopts β -a pleated sheet-like shape resembling a folded film [32].

Aβ is formed as a result of a sequence of proteolytic cleavages of APP by a beta-secretase and a gamma-secretase [33]; APP is a type I transmembrane glycoprotein with three main isoforms resulting from alternative splicing [34]. In normal circumstances, amyloid precursor protein undergoes cleavage by gamma-secretase, which influences the secretion of a soluble molecule called sAPP and C-terminal fragment 83 (CTF83). Unlike Aβ, sAPP protects neurons against toxicity and is found at lower levels in individuals with Alzheimer's than in healthy individuals. On the other hand, when APP interacts with beta-secretase (BACE1), it generates a shorter amino-terminal fragment (sAPP β) and a longer CTF99 fragment [35]. APP CTFs are then processed by -secretase to generate Aβ, which is secreted, aggregated, and deposited in extracellular plaques, resulting in amyloid neurotic plaque development [36]. Aside from Aβ production, dysregulation of Aβ metabolism, including Aβ breakdown and transportation, contributes significantly to AD pathogenesis [37]. Microglia, which are the immunological and phagocytic cells residing in the brain, exhibit a continuous surveillance of brain tissue and exhibit responses to several pathogenic stimuli. Microglia has a complex involvement in Alzheimer's disease (AD) since it contributes to inflammation, phagocytosis (cellular ingestion), and neurodegeneration (loss of nerve cells). Upon the formation of Aβ plaques, microglia undergo activation and generate proinflammatory cytokines, resulting in a persistent state of neuroinflammation [38,39].

## 3 Molecular involvement of lncRNAs in Alzheimer's disease

### 3.1 lncRNAs mediate the regulation of neuroinflammation in AD

The initial discovery of metastasis-associated lung adenocarcinoma transcript 1(MALAT1) was made in human lung adenocarcinoma, and later investigations have demonstrated its presence in other tissue types. The lncRNA MALAT1 is pivotal in regulating several cellular processes, including proliferation, apoptosis, and autophagy [40]. The upregulation of lncRNA MALAT1 expression has been associated with the suppression of neuronal death and neuroinflammation in patients diagnosed with Alzheimer's Disease (AD). Sure, researchers have proposed that the inhibitory impact elicited by MALAT1 might potentially be associated with the suppression of microglia activation via NF-κB. Peizhi and his colleagues conducted a study in an Alzheimer's disease (AD) animal model, wherein they observed that upregulating MALAT1 expression led to a reduction in neuronal apoptosis, improved neuronal functional recovery and regeneration, and a drop in levels of IL-6 and TNF-α, while concurrently boosting IL-10 level. Additionally, it was revealed that MALAT1 can inhibit the expression of miR-125b. In the context of an Alzheimer's disease (AD) model with overexpression of MALAT1, it was shown that the expression of miR-125b was downregulated. This downregulation was associated with a decrease in the release of inflammatory cytokines [41]. Furthermore, another study found that MAGI2-AS3 indirectly regulates the expression of BACE1 by interacting with miR-374b-5p. Suppression of MAGI2-AS3 was found to diminish the harmful effects on the nervous system caused by Aβ25-35, including neurotoxicity and neuroinflammation, showing its role in controlling neuroinflammation processes in AD [42].

### 3.2 LncRNAs play an essential impact on amyloid-beta (Aβ) peptide production in AD

The amyloid-beta (Aβ) peptide is a crucial element in the advancement of AD, and several studies have emphasized its association with lncRNAs in the pathogenesis of AD [43]. For instance, a study by Yuting Ding et al. to investigate the participation and underlying molecular processes of BDNF-AS in AD showed increased amounts of BDNF-AS in the bloodstream of persons diagnosed with Alzheimer's disease. These higher levels were shown to be strongly linked to the cognitive abilities of these patients, and in vitro experiments proposed that BDNF-AS has the potential to elicit neurotoxic effects. Moreover, their further experiments revealed that the induction of BDNF-AS led to an elevation in the expression of BACE1 through the inhibition of miR-9-5p, thereby promoting the formation of amyloid plaques. The research findings revealed a significant association between increased expression of BDNF-AS and the exacerbation of cognitive loss in mice with AD, suggesting that this particular long non-coding RNA plays a role in the progression of AD(44). Furthermore, the lncRNA MAGI2-AS3 has been well acknowledged for its substantial regulatory roles in many cancer types, including lung cancer [45]. It serves as a molecular sponge, exerting an influence on the activity of miR-374b-5p and BACE1, an enzyme that plays a critical role in the degradation of Aβ protein [46]. According to recent research, it has been noted that the downregulation of MAGI2-AS3 plays a significant role in the reduction of Aβ formation by sponging miR-374b-5p in individuals affected by AD, hence suggesting its involvement in the pathogenesis of AD [47]. In a study led by Zhang and colleagues, they found that inhibiting BC1 had a protective impact on the brains of mice with AD, effectively halting the accumulation of Aβ. Consequently, the mice demonstrated enhanced memory and learning abilities. Conversely, the overexpression of exogenous BC1 had the reverse effect, leading to the deposition of Aβ peptides and impairments in memory and cognition [48].

### 3.3 LncRNAs are involved in the regulation of tau protein in AD

The tau protein is an essential component in AD development, and research has demonstrated the significant involvement of lncRNAs in the control of tau protein [49]. One example of lncRNA that has been found to have widespread expression across several kinds of mammalian cells is NEAT1. Multiple pathophysiological processes have been associated with it, including neurological diseases; previous studies have established a significant association between NEAT1 and the tau protein in AD. For instance, a survey by Ke et al. provided evidence for the involvement of NEAT1 in the accelerated progression of Alzheimer's disease, and it was found that downregulation increased the generation of P-tau, enhancing Aβ levels and generating neuronal damage. The interaction between NEAT1 and miR-107 resulted in the above outcome, whereby NEAT1 served as a molecular decoy for this specific microRNA [50]. Furthermore, NEAT1 has been linked to AD due to its role in regulating the miR-124/BACE1 signaling pathway [51]. The hyperphosphorylation of tau plays a pivotal role in the progression of AD. Recent research that was conducted to examine the influence of linc00507 on Tau phosphorylation in both an animal model of AD and a cell model (Aβ42-SH-SY5Y) found that linc00507 exhibited a significant increase in the hippocampus and cerebral cortex of transgenic mice expressing APP/PS and in AD-like SH-SY5Y cells. This elevation was associated with its ability to bind to miR-181c-5p, functioning as a competitive endogenous RNA (ceRNA). Consequently, it played a role in regulating the expression of microtubule-associated protein Tau (MAPT) and tau-tubulin kinase-1 (TTBK1). The genes MAPT (responsible for encoding the tau protein) and TTBK1 (accountable for encoding a tau kinase) were confirmed as direct targets of miR-181c-5p. Moreover, linc00507 facilitated hyperphosphorylation of the tau protein by activating the P25/P35/GSK3β signaling pathway. This activation occurred through the regulation of MAPT/TTBK1 by acting as a sponge for miR-181c-5p [52]. The findings in the above-discussed research indicate that lncRNAs play a crucial role in regulating Alzheimer's disease development through molecular ways.

### 3.4 LncRNAs crucial modulators of APP expression in AD

**Fig. 1:**
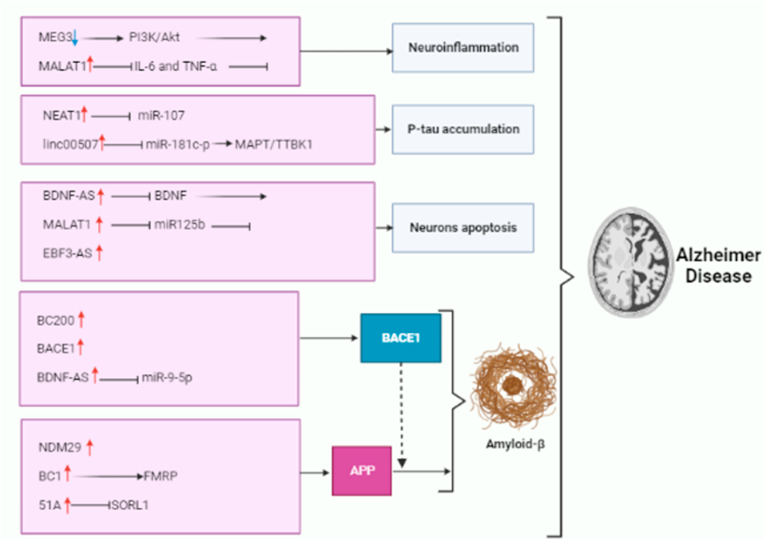
**Long non-coding RNAs molecular mechanisms involved in Alzheimer Disease:** Downregulation of MEG3 increases neuroinflammation through PI3/Akt pathway. The increased expression of MALAT1 reduces neuroinflammation by lowering IL-6 and TNF-. NEAT1 and linc00507 have been identified as Tau proteins. Increased expression of NEAT1 increased Tau protein phosphorylation by sponging miR-107, whilst increased linc00507 levels increase Tau protein hyperphosphorylation via modulating the miR-181c-5p/MAPT/TTBK1 pathway. MALAT1 upregulation increased cell death via targeting miR-125b. Furthermore, EBF3-AS is involved in the regulation of neuron apoptosis. BACE1 activity is linked to BACE1-AS, BC200, and BDNF-AS overexpression. Elevated 51A levels in AD patients changed the splicing mode of SORL1, causing harm to APP processing and promoting Aβ accumulation. BC1 upregulation promotes APP mRNA translation via binding to FMRP. NDM29 overexpression promotes APP production.

NDM29 is an example of a long non-coding RNA transcribed by the enzyme RNA polymerase III. According to existing research, NDM29 has been found to significantly impact the increase of APP levels, enhancing the production of the two main isoforms of amyloid beta (Aβ) [53]. Numerous studies have indicated that NDM29, a lncRNA, facilitates the amyloid precursor protein (APP) generation and degradation processes by activating the β-secretase and BACE1 pathways. In addition, the progression of the process can be slowed down by administering anti-inflammatory drugs, while the initiation of inflammation tends to accelerate it [54]. On the other hand, in another study, it has been found that SORL1 collaborates with APP, influencing transportation and proteolysis pathways, thereby contributing to the advancement of Alzheimer's disease [55]. Moreover, a decline in SORL1 levels results in altered processing of APP, favoring the β-secretase degradation pathway over the alternate transcriptional cycle. This shift enhances the release of APP and, consequently, the synthesis of Aβ [56]. In a separate study, researchers identified a non-coding RNA called 51A in an antisense orientation within intron 1 of the SORL1 gene. They found that the expression of 51A led to a modification in SORL1's splicing process, diverting it from generating the typical long protein variant A to an alternatively spliced protein form. The splicing alteration led to reduced production of SORL1 variant A and was associated with compromised amyloid precursor protein (APP) metabolism. Disrupted APP processing results in elevated Aβ generation [57]. The above study's findings show how lncRNAs play an essential role in the modulation of APP expression, which is a critical factor in AD progression. Overall, lncRNAs have been related to many facets of AD development, including amyloid plaque development, neurofibrillary tangle formation, neuroinflammation, synaptic dysfunction, and neuronal cell death; the overall molecular mechanisms of lncRNAs in AD are presented in (Fig. 1) and Table 1, respectively.

**Table (1):** LncRNAs implicated in AD molecular processes.

LncRNA	Regulation status	Outcomes	References
MALAT1	Upregulated	Suppression of neuronal death and neuroinflammation in AD patients	[40]
BDNF-AS	Upregulated	BDNF-AS contributes to an increase in the expression of BACE1 through the inhibition of miR-9-5p, promoting the formation of amyloid	[44]
MAGI2-AS3	Downregulated	Decreased the formation of Aβ plaque	[47]
NEAT1	Downregulated	Reduced Aβ-induced inhibition and increased apoptosis and p-Tau levels.	[50]
BC1	Overexpressed	The accumulation of Aβ peptides leads to impairments in memory and spatial learning.	[48]
NDM29	Upregulated	Increases APP synthesis and leads to the increase of Aβ secretion	[54]
SORL1	Downregulated	A decline in Its levels results in reduced production of APP	[55]
linc00507	Upregulated	Linc00507 increased tau protein phosphorylation by activating the P25/P35/GSK3β signaling pathway, regulated by MAPT/TTBK1 sequestering miR-181c-5p.	[52]

## 4 LncRNAs are ideal candidates for new AD biomarkers

Currently, the diagnosis of AD involves the utilization of neuropsychological testing. In addition to these tests, clinical diagnosis requires the examination of neuroimaging results and the monitoring of established biomarkers like the concentration of Aβ peptides (specifically, the Aβ1-42: Aβ 1-40 ratio) and the levels of total and hyper-phosphorylated proteins (Thr181 and Thr231) in CSF. PET using 1F-florbetapir or 11C Pittsburgh compound B is employed for imaging purposes; amyloid oligomers and plaque development can also be seen; however, there is still a problem with the nonlinear relationship between the concentration of amyloid in the CSF and the results of PET scans. The CSF sample, on the other hand, is quite intrusive and not always acceptable or practical in certain senior citizens. Fludeoxyglucose PET, a non-invasive imaging technique that provides data on neuronal function, is incredibly helpful in therapeutic contexts.

Magnetic resonance imaging (MRI) has shown to be an effective non-invasive tool for assessing functional deficits. MRI identifies and diagnoses amyloid plaques by improving detection capabilities using iron oxide nanoparticles (NPs) as contrast agents or fluorescent probes [58]. According to reports, these iron oxide NPs help with imaging by binding to the N terminal of Aβ.

As previously stated, reports indicate the involvement of lncRNAs in the etiology of neurodegenerative disorders. A hypothesis also suggests that plasma lncRNAs might be potential biomarkers for Alzheimer's disease (AD). The word "biomarker" refers to a broad subclass of medical signals that may be evaluated reliably and reproducibly - objective indications of medical status perceived outside the patient [59]. A biomarker may predict how effectively the body will react to treatment for an illness or condition [59,60]. Aβ plaques, a defining AD feature, are partly formed by the protein BACE1. In 2018, research found that Alzheimer's patients' plasma levels of lncRNA BACE1 were considerably more significant than those of healthy controls. Additionally, the researchers found a correlation between lncRNA BACE1 levels and the severity of cognitive impairment in Alzheimer's patients. These findings suggested that plasma lncRNA BACE1 levels might serve as an important biomarker for the identification of AD. Nevertheless, further research is necessary to confirm those results and create a valuable instrument for determining blood levels of lncRNA BACE1 [61].

Additionally, 2022 research discovered that late-onset AD victims had significantly higher plasma levels of the lncRNA BC200 and lncRNA NEAT1 than healthy controls. The researchers also found that the degree of cognitive impairment was correlated with the levels of lncRNA BC200 and NEAT1 in individuals with Alzheimer's disease; in concluding their study, they noted that plasma levels of BC200 and NEAT1 may be helpful to be used as biomarkers for identifying and tracking the progression of late-onset AD [62]. On the other hand, in another significant study, BACE1-AS expression was lower in the pre-AD subgroup but higher in the AD group than in individuals who did not have AD. A fascinating ROC curve analysis showed that lncRNA BACE1-AS might be able to differentiate between pre-AD and full-AD groups, implying that BACE1-AS may be an AD biomarker [63]. Also, Y. Deng and his colleagues discovered that lncRNA 51A was increased in the plasma of Alzheimer's patients compared to controls; they noted that lncRNA 51A was stable in plasma. Importantly, lncRNA 51A was shown to be inversely linked with Mini-Mental State Examination scores in AD patients. Their findings suggest that plasma lncRNA 51A might be a valuable biomarker for Alzheimer's disease diagnosis [64]. It was found that MMSE score reduction in AD patients at one year, two years, and three years may be predicted by CSF levels of lnc-MALAT1 and miR-125b. Still, according to Jingcong Zhuang et al., not plasma levels, lnc-MALAT1 and its target miR-125b may help treat AD because of their interactions with FOXQ1, PTGS2, and CDK5. This research showed that lnc-MALAT1 is a possible candidate for AD biomarker [65]. According to the findings of the initial studies, long non-coding RNAs are exciting possibilities for diagnostic or prognostic biomarkers for the early detection of Alzheimer's disease. It should be noted that while additional study is needed to comprehend their importance as disease biomarkers fully, their significance in disease diagnosis or universality needs to be proven for early detection purposes.

## 5 LncRNAs and immune responses in Alzheimer's disease

LncRNAs contribute to the immune response in different diseases AD, with their unique characteristic of not encoding proteins but instead regulating gene expression and other cellular mechanisms. These lncRNAs play significant roles in diverse immune responses associated with AD, including inflammation, phagocytosis, and antigen presentation [66]. For example, long non-coding RNAs have been reported to be involved in the different immune responses in various types of cancer, such as lung cancer [67], pancreatic cancer [68], and colorectal cancer [69].

In AD, several lncRNAs such as BC200 [70], MALAT1 [71], lNEAT1 [72] BACE1-AS [67] have linked to AD immune responses [73]. Innate immunity is the body's first line of defense against infection [74], and it is characterized by the release of inflammatory cytokines and the activation of immune cells such as microglia and astrocytes [75].On the other hand, adaptive immunity is the body's second line of defense against infection and is characterized by the production of antibodies and the activation of T cells [76]. As mentioned earlier, lncRNAs have been linked to AD innate immune response; for example, lncRNA NEAT1 has been shown to increase the production of inflammatory cytokines, including IL-1 and TNF- in microglia. Microglia are native immune cells in the brain that assist in cleaning up cellular debris and pathogens in Alzheimer's disease; thus, when microglia become activated, they produce an excess of inflammatory cytokines, which may kill neurons while also adding to the disease's development [72]. Furthermore, lncRNAs have also been linked to the adaptive immune response to Alzheimer's disease; for instance, the lncRNA BACE1-AS has been demonstrated to boost antibody production towards (Aβ) [77,78], a protein suspected to function in the progression of Alzheimer's disease. Additionally, separate research has shown that while BC200 is mostly recognized for its involvement in neurons, it might impact immune responses in Alzheimer's disease by indirectly influencing neuronal function and neuroinflammation [69,79]. Conversely, lncRNA MALAT1 has been associated with neuroinflammation and has been shown to regulate microglial activation, stimulating immunological responses in Alzheimer's disease (AD) [80].LncRNA research is a fast-evolving area, and more has to be found about the involvement of lncRNAs in Alzheimer's disease. However, the prospects for lncRNAs in Alzheimer's disease research are promising, and lncRNAs may ultimately lead to novel approaches to detecting, curing, and preventing this terrible illness. There is a need for further research to show the connection between lncRNAs and the immune responses in AD; this will assist in fully comprehending the role of long non-coding RNA processes in immune responses that are associated with AD and also in developing immunotherapies that can be used to cure AD.

## 6 LncRNAs as novel treatments for AD

Several studies have recently indicated that lncRNAs are implicated in amyloid formation, Tau hyperphosphorylation, mitochondrial dysfunction, synaptic impairment, and neuroinflammation, crucial to AD development. Consequently, several studies have emphasized the importance of lncRNAs in developing therapeutic targets and treatments for AD and related disorders [81]. For example, restricting BC200 boosted cell survival and reduced cell death by targeting BACE1, a process that may be aided by BC200 upregulation; the action, as previously stated, indicates that BC200 might be an effective target in the progression of AD and offers fresh perspectives into AD genetic target therapy [82].

NEAT1, a long non-coding RNA, on the other hand, has been demonstrated to have increased expression in SH-SY5Y and SK-N-SH cells treated with Aβ [83]. Notably, NEAT1 inhibition decreased the Aβ-induced reduction of cell viability, apoptosis, and p-Tau levels. NEAT1 was revealed to be a sponge for the microRNA miR-107. The researchers identified a decrease in miR-107 expression in amyloid beta-treated cells, and boosting its levels by overexpression mitigated Aβ 's harmful effects. In addition, interacting with miR-107 diminished NEAT1's neuroprotective capabilities in SH-SY5Y and SK-N-SH cells against induced neuronal damage. These findings suggest that targeting the NEAT1-miR-107 link might be a promising avenue for AD treatment, resulting in less Aβ-induced neuronal injury and greater neuroprotection. Nevertheless, further research is required to properly emphasize the complex biochemical pathways involved and determine the full therapeutic potential of this unique technique [83].

In both in vivo and in vitro Alzheimer's disease (AD) models, Du Yue et al. reported a significant increase in the levels of the lncRNA RNA XIST. Anti-miR-124 oligodeoxyribonucleotide (AMO-124) cotransfection of N2a cells lowered miR-124 negative regulation while increasing BACE1 positive modulation. Also, decreasing the lncRNA XIST reduced the impact of H2O2 on the expression of miR-124, BACE1, and A1-42 in N2a cells, which was inhibited by cotransfection with AMO-124. By lowering lncRNA XIST via miR-124, AD-related alterations in BACE1 were reduced. These findings suggest that the lncRNA XIST might be used as a therapeutic target for AD. Nonetheless, further research is required to fully comprehend the fundamental processes and assess the therapeutic effects of targeting XIST in the context of AD [84].

Another study discovered that increasing the expression of MALAT1 or CNR1 had a favorable impact on the discharge of pro-inflammatory factors IL-6 and TNF- while increasing the production of anti-inflammatory factor IL-10. When miR-30b levels were elevated, these beneficial impacts were lost. MALAT1 appears to be a miR-30b sponge, blocking it from lowering CNR1 expression. This demonstrates that the miR-30b/CNR1 network mediates the observed benefits. Furthermore, overexpression of MALAT1 or CNR1 elevated phosphorylation of PI3K (phosphoinositide 3-kinase) and AKT, two essential components of the PI3K/AKT signaling pathway. Their findings suggest that MALAT1 may help neuronal regeneration in AD by activating the PI3K/AKT signaling pathway and the miR-30b/CNR1 network. These results offer insight into future AD therapies that may boost neural regrowth and avoid neuronal degeneration [85].

On top of that, Lu Zhang et al. demonstrated that blocking SOX21-AS1 in AD mice lowered oxidative stress and neuronal death. It also caused an increase in FZD3/5 protein levels and an induction of the Wnt signaling pathway. SOX21-AS1, a long non-coding RNA that causes oxidative stress and neuronal death, has been linked to AD. FZD3/5 is a protein involved in the Wnt signaling pathway, essential for cell growth, differentiation, and survival. These findings suggest that suppressing SOX21-AS1 could represent a potential treatment method for AD [86]. According to recent research, inhibiting MIR600HG increased PINK1 levels while blocking its degradation mediated by NEDD4L. This, in turn, resulted in enhanced mitochondrial activity and a slowing of cognitive impairment in Alzheimer's disease mice. NEDD4L is a protein that participates in the ubiquitination and degradation of other proteins, while PINK1 is essential for mitochondrial repair. The production of amyloid beta plaques and tau protein tangles in Alzheimer's disease can potentially disrupt mitochondria. As a result, PINK1 degradation can occur, impairing mitochondrial activity and leading to cognitive impairment. These results show that targeting MIR600HG may provide an innovative treatment method for Alzheimer's disease [87]. So far, research indicates that lncRNAs show significant promise as future Alzheimer's disease therapeutic possibilities. To find and validate the involvement of lncRNAs in Alzheimer's disease therapy, further research is needed, such as research on Antisense oligonucleotides, which are short, single-stranded DNA or RNA molecules that are designed to bind to complementary target Antisense oligonucleotides (ASOs) belongs to a category of molecular therapeutics that exert their regulatory effects at the genetic level [88]. These ASOs are designed to attach to the DNA or mRNA of target genes selectively, therefore impeding the expression of these genes [89]. Over the last decade, therapies associated with ASO have resulted in noteworthy advancements in clinical practice. The Food and Drug Administration (FDA) has approved several antisense oligonucleotide (ASO) medications that are now available in the market for the treatment of neurodegenerative illnesses [90]. However, it should be noted that ASO, a specialized kind of nucleic acid medication, also has a significant off-target impact. Hence, despite several pre-clinical applications of targeted long lncRNAs, more investigation is essential to get enhanced outcomes., including in-depth investigations and well-designed clinical trials on how ASOs can be used to treat AD together with lncRNAs.

## 7 Conclusions

Early detection of AD presents challenges, but early detection allows early intervention to postpone or slow the degenerative process potentially. As a result, developing specific and reliable biomarkers for the early stages of Alzheimer's disease holds enormous promise for effective detection. Circulating lncRNAs found in blood, plasma, and CSF are appealing as diagnostic biomarkers due to their ease of detection using simple assays and ability to persist during storage and handling. Simultaneously, numerous investigations indicate that lncRNAs play a crucial role in governing gene expression and influencing cell growth, proliferation, apoptosis, and cellular communication. Recent studies reveal varying levels of expression changes in lncRNAs across tumors of diverse origins, suggesting their potential as future biomarkers for various cancer types, and some of the studies have been continued to their clinical trial stages. Additionally, technological advancements have aided researchers in understanding the significance of lncRNAs in combating cancer. These technologies have unveiled the potential of lncRNAs in administering therapies to regulate genes implicated in cancer development, with some studies progressing to clinical trials. Furthermore, comprehensive investigations are required to integrate state-of-the-art technologies like machine learning, single-cell RNA sequencing (scRNA-seq), and high-throughput screening for a more thorough understanding and characterization of lncRNA drugs. Utilizing micro- and nanofabrication in designing lncRNA drugs as carriers is essential for obtaining more dependable therapeutic and diagnostic outcomes. The identification and targeting of dysregulated long lncRNAs hold significant potential as a viable approach for therapeutic intervention in AD and other intricate disorders. RNA-based medicines, such as antisense oligonucleotides and small interfering RNAs, can manipulate the expression of particular lncRNAs, reinstating proper regulatory networks and alleviating the advancement of diseases. Furthermore, advancing small molecules capable of specifically targeting AD-associated lncRNAs may offer supplementary treatment possibilities. As our knowledge of lncRNAs biology expands, the chance of developing advanced and specific therapies for AD and other intricate illnesses also increases. This might potentially bring about a significant change in the field of medicine known as precision medicine. Moreover, additional clinical trials are essential to substantiate the efficacy of lncRNAs as potential daily therapeutic agents and biomarker tools not only for Alzheimer's disease but also for various other illnesses.

## Ethics approval and consent to participate

Not applicable.

## Consent for publication

Not applicable.

## Availability of data and materials

Not applicable.

## Funding

This research was funded by the 10.13039/100006190Key Research and Development Project of Shandong Province (2022CXGC010507) and the Horizontal Project of 10.13039/100009108Shandong University (12721728).

## CRediT authorship contribution statement

**Xiaoben Wu:** Writing – original draft, Visualization, Conceptualization. **Pengcheng Xia:** Writing – review & editing. **Lei Yang:** Writing – review & editing, Resources. **Chao Lu:** Writing – review & editing, Supervision. **Zhiming Lu:** Writing – review & editing, Supervision.

## Declaration of competing interest

Authors declare to have no competing interest.

## List of abbreviations

LncRNA: Long non-coding RNA
AD: Alzheimer's Diseases
BBB: Blood-brain barrier
APP: Amyloid precursor protein
CTF83: C-terminal fragment 83
BACE1: Beta-secretase 1
CSF: cerebrospinal fluid
MALAT1: Metastasis associated lung adenocarcinoma transcript 1
IGF2R: Insulin Like Growth Factor 2 Receptor
SLC22A2: Solute Carrier Family 22 Member 2
SLC22A3: Solute Carrier Family 22 Member 3
TRAMP: Trf4p/Air2p/Mtr4p polyadenylation
Xrn1: 5′-3′ exoribonuclease 1
UPF1: Up-frameshift protein 1
DNA: deoxyribonucleic acid
RNA: Ribonucleic acid
BDNF-AS: Brain-derived neurotrophic factor-antisense
EBF3: EBF Transcription Factor 3
NDM29: Neuroblastoma Differentiation Marker 29
PET: Positron emission tomography
MRI: Magnetic resonance imaging
AUC: Area under the curve
NEAT1: Nuclear enriched abundant transcript 1
BC200: Brain cytoplasmic 200
FOXQ1: Forkhead Box Q1
PTGS2: Prostaglandin-Endoperoxide Synthase 2
CNR1: Cannabinoid Receptor 1
NEDD4L: Neural precursor cell expressed developmentally downregulated 4-like
PINK1: PTEN-induced kinase 1
